# Complete Mitochondrial Genomes of Spotted Forest Musk Deer (*Moschus berezovskii*) from Huanglong Mountain, Shaanxi, China, and Phylogenetic Analysis of Moschidae

**DOI:** 10.3390/biology14121794

**Published:** 2025-12-16

**Authors:** Kuo Sun, Xiao Tan, Lei Zhang, Ying Dai, Kun Bian, Feiran Li, Lijuan Suo, Xiaojuan Du, Chao Yang, Jie Tang

**Affiliations:** 1 Shaanxi Key Laboratory of Qinling Ecological Security, Shaanxi Institute of Zoology, Xi’an 710032, China; sunkuo@snnu.edu.cn (K.S.);; 2 Shaanxi Provincial Field Observation & Research Station for Golden Monkey, Giant Panda and Biodiversity, Xi’an 723400, China; 3 School of Life Sciences, Shaanxi Normal University, Xi’an 710062, China; 4 Baoji City Forest Musk Engineering Technology Research Center, Baoji 721700, China; 5 Foping County Forest Musk Expert Workstation, Hanzhong 723400, China

**Keywords:** mitogenome, *Moschus berezovskii*, *Moschus moschiferus*, mitogenomic phylogeny, Moschidae

## Abstract

Musk deer (Moschidae), a primitive lineage within Ruminantia, are distributed across East Asia and have long been of interest in molecular phylogenetic research. The spotted forest musk deer from Huanglong (HL) Mountain in Shaanxi, China, has long been morphologically classified as *Moschus moschiferus* (Siberian musk deer). However, its true taxonomic status has remained uncertain due to the lack of comprehensive molecular evidence. Moreover, studies on mitochondrial genome (mitogenome) variation within Moschidae, particularly at the intraspecific level, remain limited. To date, few phylogenetic analyses of Moschidae have incorporated all available complete mitochondrial genomes from public databases. In this study, we sequenced and assembled the complete mitogenomes of two spotted musk deer individuals from Huanglong Mountain, Shaanxi, China, and identified them as *M. berezovskii* (forest musk deer) by phylogenetic analysis. All available complete mitochondrial genomes of Moschidae were also included in the phylogenetic reconstruction. The average complete mitogenome length of *M. berezovskii* distributed in HL was 16,355 bp, and comprises 13 protein-coding genes (PCGs), two ribosomal RNA genes, and 22 transfer RNA genes. Phylogenetic analysis and genetic distance indicate that the taxonomic identity of the HL Mountain population as *M. berezovskii* supports the monophyly of *M. berezovskii* and provides a robust phylogenetic framework for clarifying evolutionary relationships within the family.

## Simple Summary

Musk deer are small, shy animals that live in the mountains and forests of East Asia. They are famous for producing musk, a valuable substance used in traditional medicine and perfumes. However, the number of wild musk deer has dropped sharply due to illegal hunting and habitat loss. One particular population from Huanglong Mountain in Shaanxi, China, has long been considered to belong to the Siberian musk deer (*Moschus moschiferus*) due to its spotted appearance. In this study, we used the complete mitochondrial genome to clarify the taxonomic position of Shaanxi musk deer. By comparing all available mitochondrial genomes of musk deer species, we found that the Huanglong population is genetically closer to the forest musk deer (*Moschus berezovskii*) than to the Siberian musk deer. These results provide new genetic evidence for understanding species boundaries within musk deer and offer a valuable molecular basis for future research on their evolution, taxonomy, and conservation management.

## 1. Introduction

Musk deer are ruminant mammals belonging to Moschidae within the order Artiodactyla. Musk secreted by male musk deer is widely used in traditional Chinese medicine and perfume [1,2]. As the sole genus in Moschidae, *Moschus* comprises seven recognised species [3]. Some researchers recognize six species in Moschidae [4]. Musk deer are primarily distributed across the Palearctic region, with a range extending from the Himalayas to parts of Central and East Asia [2]. Recent years have witnessed a sharp decline in wild forest musk deer populations, leading to their classification as an Endangered species on the IUCN Red List [5]. In addition, the Red List of China’s Vertebrates categorizes the species as Critically Endangered [6].

To date, the geographical distribution of various musk deer species has been mainly determined [4,7,8]; however, the distribution of the spotted musk deer species in the Huanglong (HL) mountain region remains controversial. Huanglong Mountain, situated in the forested Min Mountain range at the eastern edge of the Tibetan Plateau, Shaanxi Province, China, has recently become a focal area for musk deer research [9].

**Figure 1:**
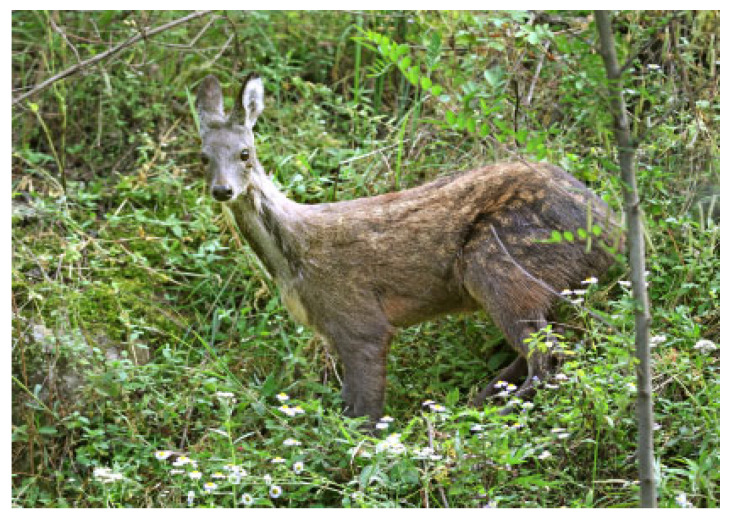
Image of a spotted forest musk deer (*M. berezovskii*) from the same population distributed in Huanglong Mountain, identified here as *M. berezovskii* based on its mitogenome. The image was taken by the team member Chao Yang in Huanglong Mountain, Minshan range, Shaanxi, China.

Since 2011, the musk deer species distributed in the HL mountain, initially identified as *Moschus moschiferus* (Siberian musk deer) based on its distinctive morphological features, including characteristic spots on its back and skull, has been reported (Figure 1) [9]. However, the taxonomic status of this population remains controversial [10,11], as previous distribution records for *M. moschiferus* in this area are lacking, and the identification was based solely on external characteristics without molecular validation [9]. The most recent mitochondrial single-gene study indicated that the musk deer distributed in HL Mountain region belongs to *Moschus berezovskii* [10]. Additionally, within Moschidae, complete mitochondrial genomes representing seven species are all available in NCBI. Nevertheless, no study to date has focused on the HL Mountain population using complete mitochondrial genome data, leaving its phylogenetic status unresolved.

Additionally, phylogenetic relationships in Moschidae have been controversial until now. For example, some studies indicate that *Moschus berezovskii* and *Moschus chrysogaster* (alpine musk deer) were not monophyletic [12]. The latest research suggests that *Moschus berezovskii* population in Shanxi Province had significant genetic differentiation [11].

In other animals, complete mitogenomes have been used to reconstruct phylogenetic trees and evaluate lineage divergence. For instance, in Tibetan argali (*Ovis ammon hodgsoni*), complete mitogenomes resolved phylogenetic relationships within Tibetan argalis and clarified the relationship within *O. ammon* subspecies [13]. Similarly, for *Camelus dromedarius* (wild dromedary camels), the mitogenome has been applied across multiple populations of *C. dromedarius* and related species to reconstruct phylogenies, assess species-wise genetic distinction and detect gene-level signals of selection that likely reflect adaptive divergence among lineages in different environments [14]. These examples illustrate that complete mitogenomes provide high-resolution phylogenetic signal, outperforming analyses based on partial mtDNA or single genes.

In this study, we sequenced and assembled two complete mitochondrial genomes of wild forest musk deer from HL Mountain, Shaanxi, China. To clarify phylogenetic relationships within Moschidae, we further assessed intraspecific variation by comparing mitogenomes from multiple individuals. To address the current lack of a comprehensive phylogenetic framework for Moschidae based on complete mitochondrial genomes, we reconstructed a phylogeny using all available mitogenomes from GenBank (as of 6 June 2025). This study provides a robust foundation for understanding the evolutionary relationships and diversification of musk deer.

## 2. Materials and Methods

### 2.1. Specimen Collection and DNA Extraction

Hair samples were collected from two wild adult musk deer individuals in HL Mountain, Shaanxi Province, from 2018 to 2020 during rescue operations (Table 1). Samples were stored at Shaanxi Institute of Zoology, Xi’an, China (contacts: Chao Yang, chaoy819@xab.ac.cn). Total genomic DNA was extracted using the DNeasy Blood and Tissue Kit (Qiagen, Hilden, Germany) following the manufacturer’s protocol.

### 2.2. Mitogenome Sequencing

The complete mitochondrial genomes of two *M. berezovskii* individuals were obtained through amplification of partially overlapping PCR fragments. A total of thirty-two fragments were generated using mammal universal primer pairs suggested by Hassanin et al. (2009), which were used for Sub PCRs [15]. Each PCR reaction was carried out in a 50 μL mixture containing 25 μL of 2× Taq PCR StarMix with loading Dye (GenStar, Beijing, China), 3 μL of each primer (10 μM, forward and reverse), 8 μL of template DNA, and 11 μL of sterile double-distilled water to reach the final volume.

PCR amplification was carried out on an Applied Biosystems 2720 thermal cycler with a heated lid (Applied Biosystems, Foster City, CA, USA). The PCR protocol included an initial denaturation at 95 °C for 5 min, followed by 35 cycles of denaturation at 95 °C for 30 s, annealing at 50–55 °C for 30 s, and extension at 72 °C for 1 min. A final extension step was performed at 72 °C for 8 min, after which samples were held at 4 °C. When necessary, the annealing temperature was lowered from 55 °C to 50 °C to improve amplification efficiency and specificity. Amplification products were visualised on 1% agarose gels stained with ethidium bromide, purified using Wizard PCR preps DNA Purification System (Promega, Madison, WI, USA), Promega gel extraction kit, and subsequently sequenced in both directions with the same primer sets on an ABI 377 DNA sequencer (Applied Biosystems, Foster City, CA, USA).

### 2.3. Mitogenome Assembly and Annotation

Mitogenome assemblies were carried out in Geneious Prime (v2022.1.1) following the overlap–layout–consensus (OLC) strategy [16]. Briefly, sequence overlaps (O) were first detected among reads. An assembly layout (L) was constructed based on these overlaps, and a consensus (C) sequence was subsequently generated from the layout. Several rounds of mapping were performed, starting with an initial alignment to a reference mitochondrial genome and subsequently extending the contigs by iterative mappings to the progressively elongated sequence. For assembly guidance, the mitogenome of *Moschus berezovskii* (GenBank accession: NC_012694) was used as a reference [17].

**Figure 2:**
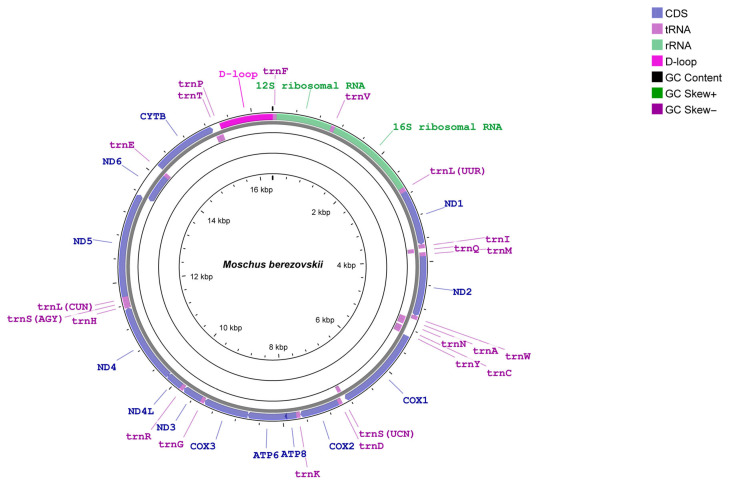
Mitochondrial genome map of spotted forest musk deer, *M. berezovskii*. The genes in the outermost circle are transcribed clockwise, and the genes in the inner circle are transcribed counterclockwise. GC-skews and GC content are shown in the inner circles. The mitogenome of a second individual was identical in gene order and orientation.

Annotation of protein-coding genes, tRNAs, and rRNAs was conducted using MITOS2 [18], with the reference dataset set to Metazoa and the vertebrate mitochondrial genetic code applied. All annotations were further curated manually within Geneious Prime (v2022.1.1) [16]. The mitochondrial genome maps were visualised using Proksee v1.0.0 (https://proksee.ca/, accessed on 29 June 2025) (Figure 2).

### 2.4. Comparative Mitogenomic Analyses

We calculated nucleotide compositional asymmetry of all available musk deer using the following formulas: AT-skew = (A − T)/(A + T) and GC-skew = (G − C)/(G + C). PhyloSuite v1.2.3 was employed to determine nucleotide composition and relative synonymous codon usage (RSCU) [19]. For each protein-coding gene (PCG) within Moschidae, synonymous (Ks) and nonsynonymous (Ka) substitution rates were estimated using the kaks function in the R package seqinr v4.2-36 [20], while nucleotide diversity (pi) was calculated via nuc.div function in pegas [21]. Genetic distances were calculated using the K2p model via the dist.dna function of R package ape v5.8-1. The K2p model was selected because it accounts for differences between transition and transversion substitution rates while maintaining a relatively simple parameterisation, which makes it particularly suitable for datasets characterised by low to moderate sequence divergence [22,23].

### 2.5. Mitogenomic Phylogeny and Divergence Time Estimation

**Figure 3:**
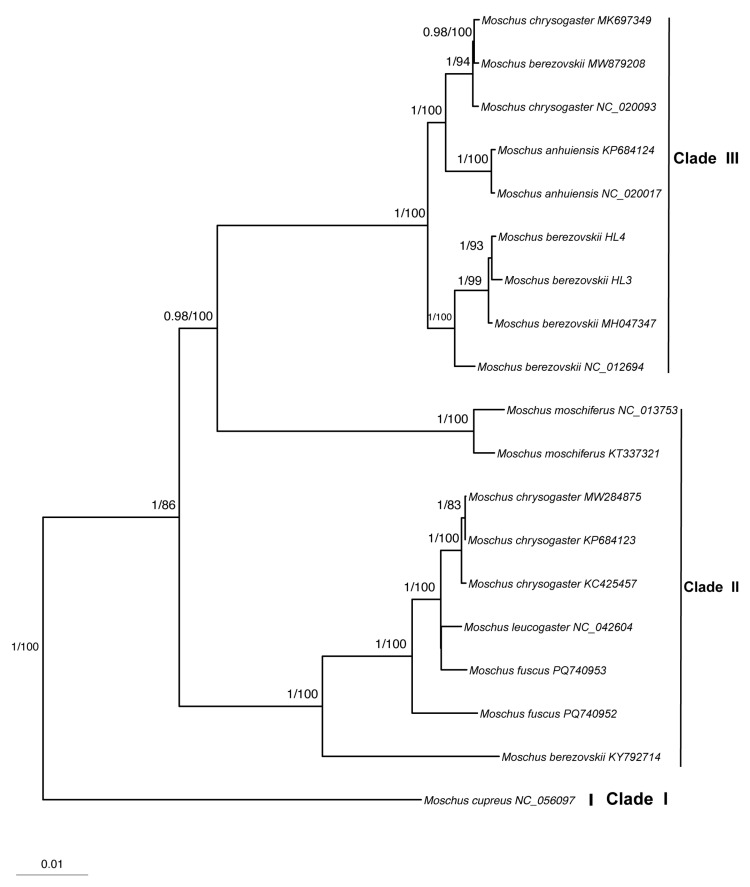
The BI and ML phylogenetic tree of seven Moschidae species based on mitochondrial 13 CDS. Numbers above the branches represent posterior probability (BI) and bootstrap value (ML), respectively. This tree includes only *Moschus* species for clarity; the complete tree with all outgroup taxa is provided in Supplementary Data S1.

**Figure 4:**
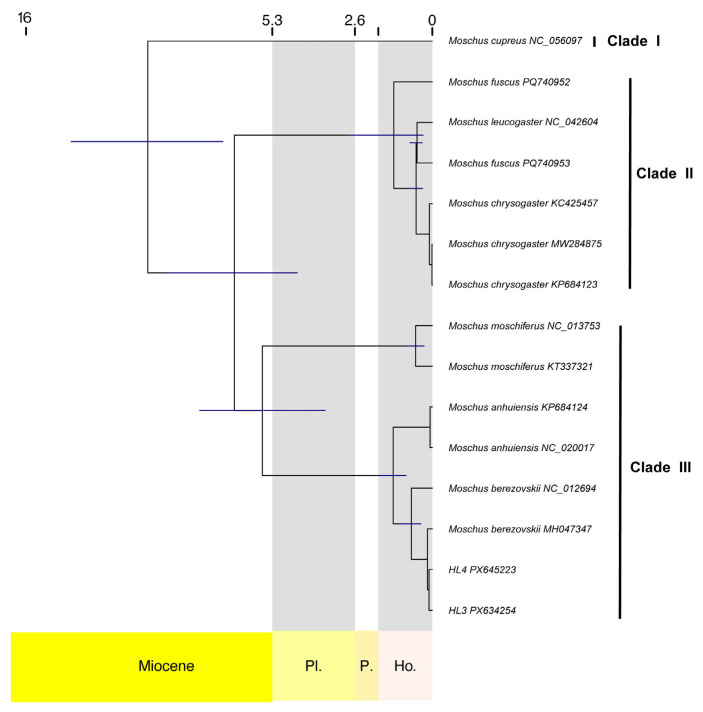
Time-calibrated phylogenetic tree of Moschidae species inferred from the 13 mitochondrial protein-coding genes, with four sequences (MW879208, NC020093, MK697349, and KY792714) removed for clarity. The complete time-calibrated phylogeny, including all sequences and outgroups, is provided in Supplementary Data S2.

We first searched for and downloaded all available mitochondrial genome sequences of musk deer and selected representative ruminant species from the NCBI database. To improve phylogenetic accuracy and avoid potential long-branch attraction, we subsequently included additional representative species from several ruminant families (e.g., Bovidae, Cervidae, Giraffidae, and Antilocapridae). In total, 27 sequences were included in the phylogenetic analyses, comprising species from Ruminantia, and representing nine genera across six families. The accession numbers and taxonomic information for sequences used in the study are listed in Table S1. Among these, 25 sequences were retrieved from NCBI, and two were newly sequenced in this study. Following genome-scale analyses [24], which identified Tragulidae as the sister group to Pecora, *Tragulus kanchil* was designated as the outgroup. To ensure data independence, we manually checked accession numbers and excluded redundant records derived from identical individuals (e.g., multiple submissions of the same *M. berezovskii* individual sequence). The final dataset of sequences included in the phylogenetic analyses is summarized in Table S1. As the primary objective of this study was to focus on phylogenetic relationships within Moschidae, we present here only the phylogeny of Moschidae (Figure 3 and Figure 4).

The sequences of 13 mitochondrial protein-coding genes (CDS) were extracted from all 27 mitogenomes using a custom R script. Each gene was aligned with MUSCLE under default settings [25]. The concatenated dataset was partitioned by gene and codon position, and the most appropriate substitution models were determined using ModelFinder implemented in IQ-TREE v2.0.6 (option: -m MFP + MERGE) [26]. Node support was estimated using ultrafast bootstrap analysis with 1000 replicates (-B 1000) [27]. Additionally, partitioning schemes for the 13 PCGs were assessed using PartitionFinder 2 [28], considering both gene and codon-level partitions. Bayesian inference (BI) was conducted using MrBayes v3.2.7 [29], with two independent runs of four Markov chains each, for 5,000,000 generations, and trees sampled every 1000 generations. Convergence and mixing efficiency were evaluated in Tracer v1.7.1, confirming that all ESS values exceeded 200. The resulting phylogenetic trees were visualized and annotated in ggtree v3.13.0.

We estimated divergence times using MCMCTree implemented in PAML v4.9 (Yang, 2007) [30]. The maximum-likelihood topology inferred by IQ-TREE was fixed as the guide tree. Four fossil calibrations derived from previous studies (Table S2) were applied as node-age constraints [31]. Two independent MCMC runs were performed for 1,000,000 generations, sampling every 100 generations and discarding the initial 100,000 generations as burn-in. Convergence was confirmed by the agreement of posterior estimates between the two runs, and all model parameters showed effective sample sizes (ESS > 200) when assessed usingTracer v1.7.1 [32], in accordance with the recommendations of dos Reis & Yang (2019) [33].

## 3. Results

### 3.1. Huanglong Mountain Moschus berezovskii Mitogenome Structure and Organization

We assembled two complete mitochondrial genomes of *M. berezovskii* distributed in the HL mountain (Figure 1; Table 1 and Table 2). Both mitogenomes are circular, double-stranded DNA molecules, each comprising 13 protein-coding genes (PCGs), two ribosomal RNA genes (rRNAs), 22 transfer RNA genes (tRNAs), and a non-coding control region (D-loop) (Figure 2). Twelve of the 13 PCGs and 14 of the 22 tRNAs were encoded on the J-strand. In contrast, the remaining eight genes were located on the N-strand (Figure 2). In both mitogenomes, the D-loop region was positioned between tRNA Pro (P) and tRNA Phe (F).

The mitogenome lengths of the two *M. berezovskii* individuals sequenced in this study were 16,355 bp and 16,363 bp, respectively (Table 2). Excluding the D-loop region, the mitochondrial DNA sequences measured 15,439 bp and 15,431 bp, respectively, indicating D-loop sizes of 924 bp (HL3 and HL4). The gene arrangement of the two *M. berezovskii* mitogenomes was conserved and entirely consistent with that of previously sequenced *M. berezovskii* (Figure 2). No mitochondrial gene rearrangements were detected. The overall GC content of the two *M. berezovskii* mitogenomes averaged 37.75% (Table 2).

Across six *M. berezovskii* individuals, including two newly sequenced specimens from HL Mountain (HL3 and HL4), mitogenome sizes ranged from 16,351 bp (MW879208.1) to 16,363 bp (HL3). The total length of PCGs showed slight variation (11,397–11,418 bp), whereas rRNA and tRNA genes were highly conserved, spanning 2525–2532 bp and 1510–1512 bp, respectively. The overall GC content remained stable (37.7–37.9%), with comparable values in PCGs (37.3–38.3%) and rRNAs (37.4–38.7%), while tRNAs displayed relatively lower GC content (33.8–35.4%). Notably, specimen HL3 harbored the longest mitogenome (16,363 bp) and the lowest GC content in tRNAs (33.8%), whereas the remaining individuals exhibited highly conserved features.

### 3.2. Codon Usage and Comparative Mitogenomic

*M. berezovskii* had the same start and stop codons for 13 PCGs. However, there was a significant variation in the stop codons of the 13 PCGs in *M. berezovskii*. The stop codons of COX1, COX2, ATP8, ATP6, ND4L, and ND5 were TAA, while ND6 terminated with TTA, and CYTB terminated with AGA. while ND1 and ND3 terminated with the truncated stop codon TA-, and ND2, COX3, ND4 terminated with T--.

In Figure S1, we show codon usage, relative synonymous codon usage (RSCU), and codon family proportions (reflecting amino acid usage) across musk deer species. Statistical analysis of RSCU indicated that the six *M. berezovskii* specimens exhibited highly consistent codon usage patterns. Among PCGs of *M. berezovskii*, Leucine1 (12.13–12.35%), Threonine (8.15–8.23%), and Alanine (6.31–6.36%) were the most frequently used amino acids, whereas Cysteine (0.54–0.58%) and Serine1 (1.42–1.44%) were relatively underrepresented (Figure S1).

Nucleotide diversity varied significantly across various mitochondrial genes (Figure S2). For COX1 and ATP8, the average nucleotide diversity was 0.0945 and 0.2037, respectively, with the proportion of variable DNA sites spanning from 33.85% in COX1 to 80.37% in ATP8 (Figure S2A). To gain a deeper understanding of the selective pressure on mitochondrial PCGs among Moschidae, the average Ka/Ks ratio for each PCG was calculated and compared (Figure S2B). The Ka/Ks ratio of 1 indicates neutral mutations, the Ka/Ks ratio of less than 1 indicates negative selection, and the Ka/Ks ratio of greater than 1 indicates positive selection [30]. All PCGs in Moschidae mitogenomes showed Ka/Ks ratios < 1. Among them, ATP8 exhibited the highest Ka/Ks ratio (0.24), whereas COX1 showed the lowest (0.023) (Figure S2B).

Pairwise genetic distances among all musk deer specimens were visualised in a heatmap (Figure S3). HL3 and HL4, representing *M. berezovskii*, exhibited an extremely low genetic distance between each other (0.0018). Their mean distances to *M. berezovskii* individuals ranged from 0.0015 to 0.017 after excluding one potentially misidentified sequence (KY792714). This level of variation is within the expected intraspecific range, especially considering that HL3 and HL4 show much larger distances to other *Moschus* species (up to 0.084). In contrast, the genetic distances between HL3/HL4 and *M. moschiferus* (forest musk deer, previously considered conspecific with spotted musk deer) were markedly higher (0.062–0.064), which is comparable to interspecific levels observed among recognized *Moschus* species. Distances to other species (e.g., *M. fuscus*, *M. cupreus*, *M. moschiferus*) ranged from 0.06 to 0.09. These results demonstrate that HL3 and HL4 are genetically clustered with *M. berezovskii* rather than *M. chrysogaster*.

### 3.3. Phylogenetic Reconstruction and Time Calibration

Our study represents the most comprehensive phylogenetic analysis of the family Moschidae, based solely on mitochondrial data, utilizing 13 protein-coding genes from 19 mitogenomes that represent all seven recognized species. Phylogenetic trees constructed using both maximum likelihood and Bayesian inference yielded identical topologies for Moschidae, indicating a robust dataset and reliable phylogenetic reconstruction. Using multiple ruminant species as the outgroup, our phylogenetic analyses recovered all musk deer species with high support (Figure 3; Supplementary Data S1).

Our analyses recovered all musk deer species with strong nodal support (Figure 3). Within Moschidae, *M. cupreus* consistently occupied the basal position, while the remaining species formed three well-supported clades. *M. cupreus* formed a distinct lineage, designated as Clade I, separate from the other musk deer species (Figure 3). Clade II includes *M. chrysogaster*, *M. fuscus*, and *M. leucogaster*. Within this clade, *M. fuscus* and *M. leucogaster* were recovered as sister taxa, forming a sister group to *M. chrysogaster*. Notably, *M. fuscus* was not monophyletic in our analysis, suggesting potential cryptic diversity or historical introgression in this species. Clade III comprises *M. anhuiensis*, *M. berezovskii*, and *M. moschiferus*, among which *M. anhuiensis* and *M. berezovskii* formed a sister group closely related to *M. moschiferus*. It is noteworthy that spotted musk and *M. berezovskii* were clustered together, supporting their close phylogenetic relationship. Overall, our topology reveals a significant divergence between the southern lineage (Clade II) and the northern lineage (Clade III) within Moschidae. In addition, four sequences (MW879208, NC_020093, MK697349, and KY792714) showed unexpected phylogenetic placements, suggesting that these samples may have been misidentified or represent potential hybrid individuals (Figure 3 and Figure 4).

In Moschidae, *M. cupreus* was inferred to have diverged earliest from all other species at approximately 9.74 Ma (Figure 4). The Clade II subsequently split from other Moschidae at 6.77 Ma. Within Clade III, the divergence between *M. moschiferus* and other species occurred around 5.8 Ma. The split between *M. berezovskii* and *M. anhuiensis* was estimated at approximately 1.35 Ma. The two newly sequenced HL individuals (HL3 and HL4) exhibited a very recent divergence, with their common ancestor dating to 0.11 Ma. Their split from other *M. berezovskii* mitogenomes occurred between 0.08 and 0.26 Ma.

## 4. Discussion

In this study, we sequenced the complete mitochondrial genomes of two spotted musk deer individuals from Huanglong Mountain, Shaanxi, China. The mitochondrial genome sizes of the HL *M. berezovskii* individuals (16,355 bp and 16,363 bp) were highly consistent with previously reported mitogenomes of the species, indicating a low level of structural variation within the lineage [11,17]. The slight difference in total mitochondrial genome length between HL3 individual and other *Moschus* species is attributable to a minor discrepancy in the ND4 gene (~7 bp). This variation results from a ~30 bp region of ambiguous bases in the ND4 sequence of the HL3 individual, likely causing a small assembly artifact. Although our mitogenomes were PCR-amplified, PCR-based mtDNA sequencing is known to produce uneven coverage and localized base-calling errors, which may lead to small indels or ambiguous bases [34].

Minor differences in mitogenome length and GC composition observed among *M. berezovskii* individuals may reflect subtle selective pressures acting across different environmental conditions, potentially influencing mitochondrial genome stability and function [35]. Slight shifts in GC content could be driven by regional variation in mutation rates or by selection associated with replication and transcription efficiency [36]. Such fine-scale compositional adjustments, although small, might provide insight into the adaptive and evolutionary dynamics of musk deer mitogenomes. In addition, several protein-coding genes (ND1, ND3: TA-; ND2, COX3, ND4: T--) possess incomplete stop codons. Such truncated stop codons are commonly observed in metazoan mitochondrial genomes and are typically converted into complete TAA codons through post-transcriptional polyadenylation, ensuring the proper termination of protein synthesis [37]. This pattern is consistent with observations in other vertebrate mitochondrial genomes, suggesting that the post-transcriptional modification mechanism of mitochondrial mRNAs is conserved in *M. berezovskii* [38]. The universally low Ka/Ks ratios across all mitochondrial protein-coding genes in *M. berezovskii* indicate long-term purifying selection, consistent with the strong functional constraints typical of mammalian mitogenomes [39]. For example, similar low Ka/Ks ratios have been reported in camels, reflecting pervasive purifying selection in their mitochondrial genes [14,40].

Although some previous morphological and mitochondrial studies classified the “spotted musk deer” as *M. chrysogaster*, mitogenome genetic distances provide no support for this taxonomic assignment. Instead, two individuals from HL Mountain show a level of divergence consistent with intraspecific variation within *M. berezovskii*. Despite the general concordance between our phylogenetic results and previous molecular studies, the taxonomic boundaries within musk deer remain unresolved [11,41]. Recent genomic studies revealed significant genetic differentiation among *M. berezovskii* populations from different mountain ranges in Shanxi Province [11], as well as clear divergence between the Shaanxi and Sichuan populations based on whole-genome data [42]. These findings suggest limited gene flow and possible local adaptation among geographically isolated populations, highlighting that *M. berezovskii* may represent a species complex rather than a single panmictic entity.

In our phylogenetic reconstruction, we excluded four sequences because they displayed anomalous placements that were inconsistent with those of other *Moschus* samples. Yang et al. (2021) also reported phylogenetic incongruence among captive and wild *M. berezovskii* individuals, attributing it to captive versus wild origin [43]. A study of *M. chrysogaster* has revealed significant genetic differentiation between captive and wild populations [44]. However, as more forest-musk deer sequences have accumulated, many without explicit sample information, we found no association between phylogenetic outliers and captive or wild status. Moreover, our HL individuals cluster closely with MH047347, which was previously suggested to represent a wild-origin outlier, contradicting the idea that phylogenetic discordance is driven by captivity. Therefore, we consider these outlier sequences more likely to represent misidentified individuals or possible hybrids. Similar cases have been reported, such as birds (*Phalacrocorax capillatus*) [45], skipper butterflies [46], and deer [47].

Previous morphological studies have revealed that skull variation among populations exceeds that observed between current subspecies, implying that cranial traits alone are insufficient for taxonomic delimitation [48]. Moreover, despite marked genetic differentiation between *M. berezovskii* (North China lineage) and *M. moschiferus*, their pelage coloration remains similar [9]. This pattern suggests that external morphological traits such as coat color are evolutionarily labile and may not reliably reflect species boundaries. In contrast, molecular phylogenies based on genome-scale or mitochondrial data reveal more apparent lineage divergence, providing a more accurate reflection of the evolutionary history of Moschidae. Therefore, the spotted musk deer should be considered a geographic or morphological variant of *M. berezovskii*, rather than a member of the *M. moschiferus* lineage.

Our divergence-time estimates within Moschidae are substantially older than those reported by Pan et al. 2015 (MRCA ~4.42 Ma) [4]. In our study, broader species sampling—including more *Moschus* species and multiple individuals per species—likely affords a more comprehensive view of mitogenomic variation. Increasing taxon sampling has been shown to improve the precision, and often the accuracy, of divergence-time estimates in mammals [49]. Nevertheless, our estimates are generally close to previous age inferences based on the cytochrome b gene [50]. Therefore, our deeper age estimates may better reflect a more accurate evolutionary timescale of Moschidae.

Our findings clarify the true taxonomic identity of the HL musk deer population and provide an updated phylogenetic framework for Moschidae, which has direct implications for the conservation and management of *M. berezovskii* distributed in HL, as accurate species identification is essential for defining conservation units. In the future, incorporating nuclear genomic data and broader geographic sampling will be important for resolving remaining uncertainties in species boundaries, detecting potential introgression, and improving our understanding of the evolutionary history of musk deer.

## 5. Conclusions

Musk deer previously identified as *M. moschiferus* distributed in Huanglong Mountain were genetically assigned to *M. berezovskii*, rather than to *M. moschiferus*, as suggested by earlier morphological classifications. In addition, our results support the distinctness of *M. berezovskii*, *M. moschiferus*, *M. anhuiensis*, *M. leucogaster*, *M. cupreus*, and *M. chrysogaster*, while *M. fuscus* exhibits non-monophyly. These findings underscore the need for integrative taxonomic assessments combining molecular, morphological, and ecological evidence to clarify species boundaries and evolutionary relationships in musk deer.

## Figures and Tables

**Table 1:** Details of samples used in this study.

Species	Sample ID	Collection Date	Gender	Collection Site	Altitude	Samples
*Moschus berezovskii*	HL3	7 August 2020	Male	Huanglong Mountain, Yanan, Shaanxi, China (110.15287410, 35.53150612)	1240 m	Hair
*Moschus berezovskii*	HL4	28 August 2017	Female	Huanglong Mountain, Yanan, Shaanxi, China (110.05054012, 35.50646264)	1353 m	Hair

**Table 2:** Length and GC content of different areas in six individuals of *M. berezovskii*.

Sample ID	Length (bp)				GC Content (%)			
(GenBank Accession)	Mitogenome	Protein-Coding	rRNA	tRNA	Mitogenome	Protein-Coding	rRNA	tRNA
		Genes				Genes		
*M. berezovskii4* (MH047347)	16,353	11,418	2526	1511	37.80%	37.90%	38.60%	35.40%
*M. berezovskii3* (NC_012694)	16,354	11,418	2525	1511	37.80%	37.90%	38.70%	35.30%
*M. berezovskii2* (MW879208)	16,351	11,400	2525	1511	37.90%	38.10%	38.70%	35.20%
*M. berezovskii* (KY792714)	16,353	11,397	2525	1512	37.90%	38.10%	38.40%	35.10%
HL4 (PX645223)	16,355	11,400	2528	1510	37.80%	38.30%	38.40%	35.10%
HL3 (PX634254)	16,363	11,397	2532	1512	37.70%	37.30%	37.40%	33.80%

## Data Availability

The mitochondrial genome sequences generated in this study are being submitted to the NCBI GenBank database, and accession numbers will be provided upon acceptance of the manuscript.

## Supplementary Materials

The following supporting information can be downloaded at: https://www.mdpi.com/article/10.3390/biology14121794/s1, Table S1: Taxonomic information and Genbank accession numbers for taxa used in the phylogenetic analysis. Table S2: Four Nodes used for calibrating the divergence time. Figure S1: A comparative analysis of the mitogenomes of *M. berezovskii* in terms of RSCU. Figure S2: Evolutionary rates of mitochondrial genes for 13 species from 17 Moschidae sequences. Figure S3: Genetic distance analysis of 13 CDS. The Supplementary Files in this study are provided in the files of Supplementary Data S1 and S2.
