# Supplementary material for: Complete Mitochondrial Genomes of Spotted Forest Musk Deer (Moschus berezovskii) from Huanglong Mountain, Shaanxi, China, and Phylogenetic Analysis of Moschidae
Source: Biology (Basel). 2025 Dec 16;14(12):1794. doi: 10.3390/biology14121794 (PMC12730355; doi:10.3390/biology14121794)
Supplement: Supplementary file 1 [file biology-14-01794-s001.zip › Figure S1-S3.pdf]

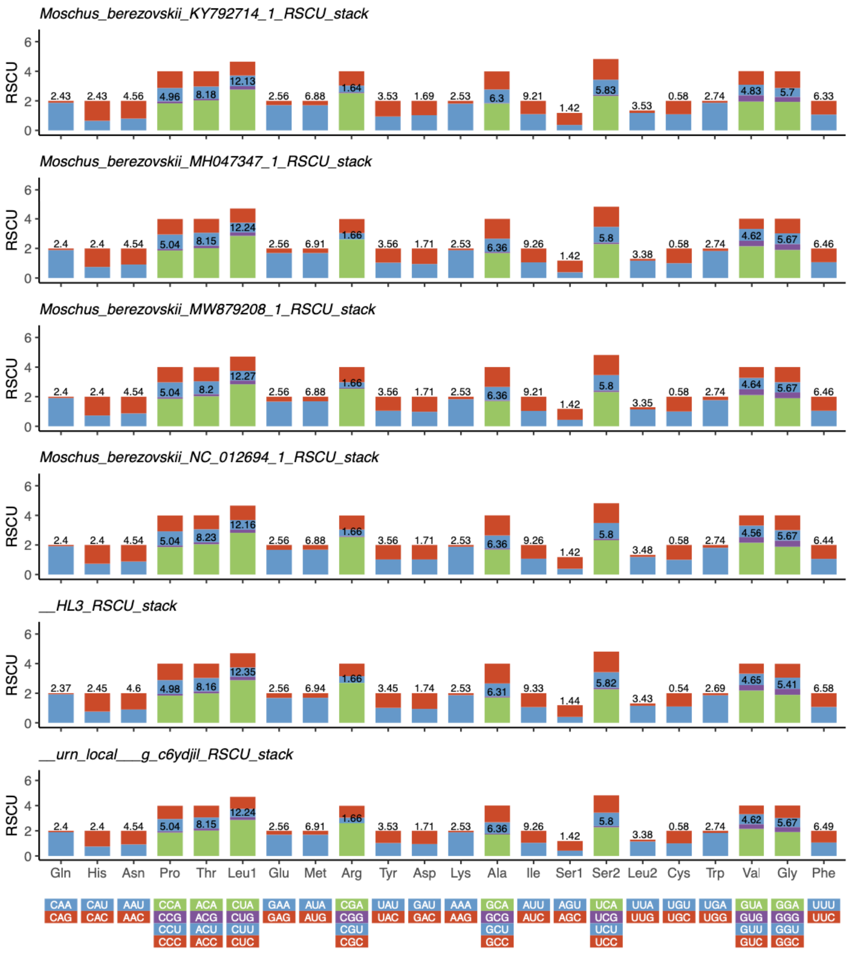

Figure S1. A comparative analysis of the mitogenomes of *M.berezovskii* in terms of RSCU.

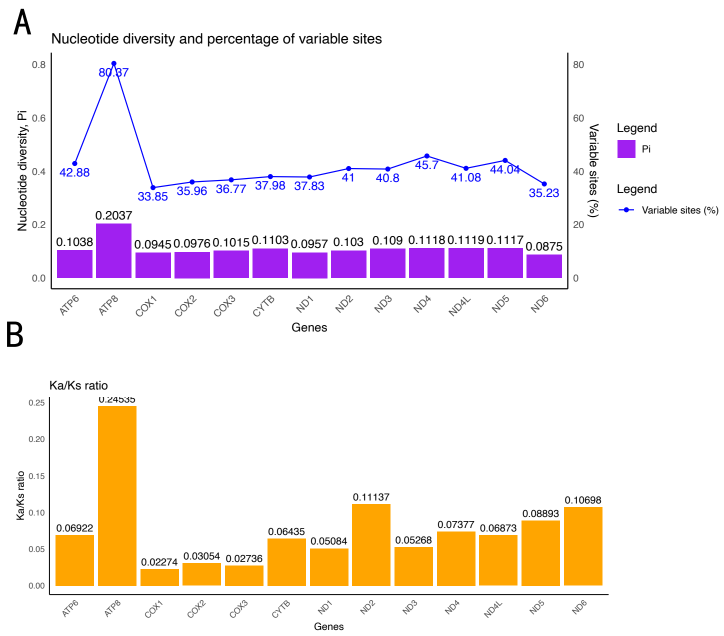

Figure S2. Evolutionary rates of mitochondrial genes for 13 species from 17 Moschidae sequences. (A) The diversity of nucleotides and the percentage of variable sites; (B) The ratio of non-synonymous substitutions to synonymous substitutions.

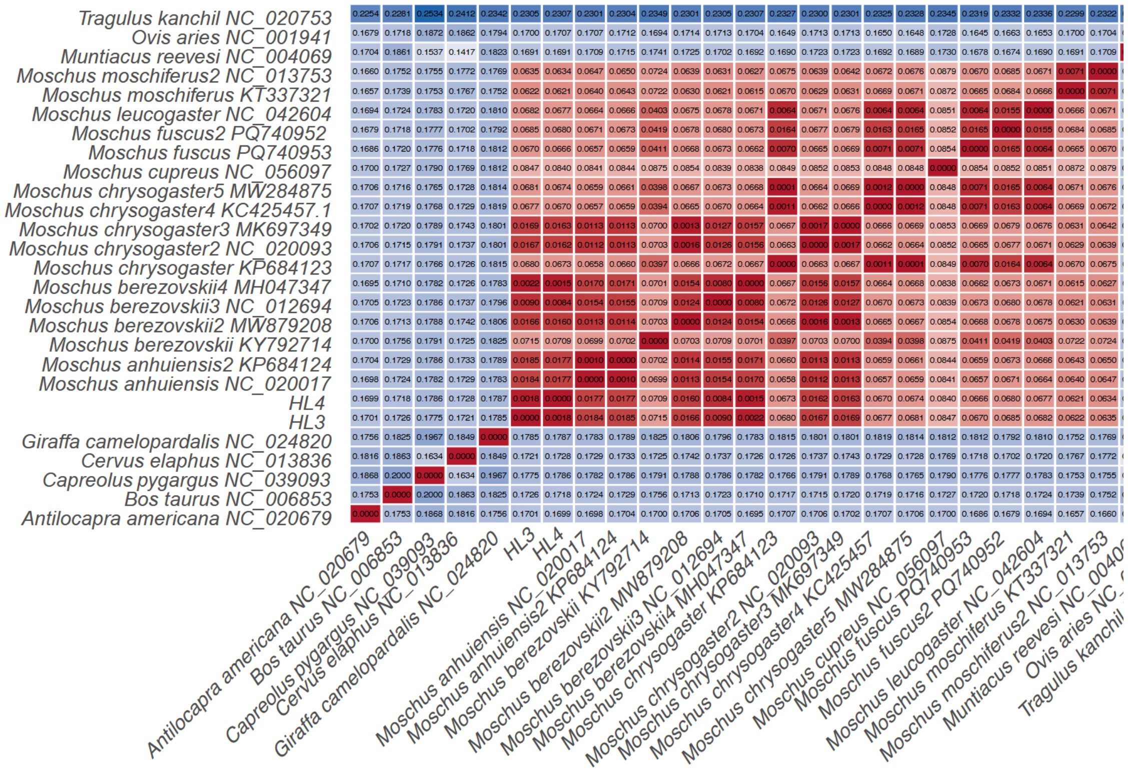

Figure S3. Genetic distance analysis of 13 CDS. The genetic distance matrix: K-2p distance, red indicated the lower genetic distance, blue indicates the higher genetic distance value.
